# MESOTIP: Phase II multicenter randomized trial evaluating the association of PIPAC and systemic chemotherapy vs. systemic chemotherapy alone as 1st-line treatment of malignant peritoneal mesothelioma

**DOI:** 10.1515/pp-2019-0010

**Published:** 2019-06-27

**Authors:** Olivia Sgarbura, Sophie Gourgou, Diego Tosi, Naoual Bakrin, Nabila Bouazza, Stéphanie Delaine, Hélène De Forges, Marc Pocard, François Quénet

**Affiliations:** Surgical oncology Department, Montpellier Cancer Institute (ICM), University of Montpellier, Montpellier, France; Biometrics Unit, Montpellier Cancer Institute (ICM), University of Montpellier, Montpellier, France; Early phase clinical trial unit, Montpellier Cancer Institute (ICM), University of Montpellier, Montpellier, France; Surgery Department, Lyon Sud University Hospital, Claude Bernard University, Lyon, France; Clinical Research Department, Montpellier Cancer Institute (ICM), Univ. Montpellier, Montpellier, France; INSERM U1275, CAP Paris-Tech, Carcinomatosis Peritoneum Paris Technology, Lariboisière Hospital, AP-HP, 2 rue Ambroise Paré – 75010 Paris – Université de Paris, France

**Keywords:** conversion to respectability, front line, intraperitoneal chemotherapy, malignant peritoneal mesothelioma, Pressurized Intra-Peritoneal Aerosol Chemotherapy (PIPAC)

## Abstract

**Background:**

Malignant peritoneal mesothelioma (MPM) is a rare tumoral disease characterized by the diffuse involvement of the peritoneal serosa. The standard frontline treatment of MPM is cytoreductive surgery with hyperthermic intraperitoneal chemotherapy (HIPEC) unless the peritoneal disease is considered unresectable. For unresectable patients the standard frontline treatment is a combination of cisplatin and pemetrexed but the prognosis remains ominous with only 13 months of overall survival (OS).

**Methods:**

The proposed study is a multicenter randomized non-comparative study evaluating the association of Pressurized Intra-Peritoneal Aerosol Chemotherapy (PIPAC) and systemic chemotherapy vs. systemic chemotherapy alone as first-line treatment of MPM. Patients will be randomized with a 2:1 ratio using a minimization technique. Sixty-six patients have to be enrolled. Stratification will be performed according to histology (epithelioid vs. sarcomatoid and biphasic), presence of extraperitoneal disease and center. Primary objective is OS and secondary objectives include progression-free survival (PFS), safety, compliance, feasibility, conversion to resectability, histological response to treatment and quality of life.

**Conclusions:**

We expect to show that intensification of the first line treatment with PIPAC for initially unresectable MPM patients increases OS.

**Trial registration:**

Prospective study. Clinicaltrials.gov: NCT03574493 EudraCT: 2019–001515-23.

## Introduction

Malignant peritoneal mesothelioma (MPM) is a rare tumoral disease characterized by the diffuse involvement of the peritoneal serosa [1]. The incidence of mesothelioma varies globally but it affects in a larger measure the industrialized countries [2, 3]. In France, the estimated incidence is 300 cases per year [3, 4, 5]. Three types of malignant mesotheliomas are described in the WHO classification: epithelioid, sarcomatoid and biphasic. The epithelioid subtype accounts for 75% of all cases and has a significantly better prognosis than the other two. Sarcomatoid subtype is extremely rare and has ominous prognosis whereas the biphasic subtype includes histological components of the other two with each contributing at least 10% of the overall histology. The biphasic subtype accounts for almost 25% of all cases and has a similar prognostic to the sarcomatoid subtype [3, 6].

The standard treatment of MPM is surgery. It has been shown that cytoreductive surgery (CRS) associated to hyperthermic intraperitoneal chemotherapy (HIPEC) improves prognosis resulting in a median overall survival (OS) of 29.5 to 53 months and a 5 years OS rate ranging between 39 and 63% [7, 8, 9]. CRS should be complete or almost complete (CCR0/1) as macroscopic residual disease deteriorates prognosis [10].

However some patients are not eligible for surgery due to the locoregional extension of the disease. Although debulking surgery may still be considered, its results are less encouraging than CRS and HIPEC.

The neoadjuvant treatment combining Cisplatin and Pemetrexed became a routinely applied option for initially unresectable patients after the publication of an open-label study [11] inspired by previous results of a randomized trial in pleural mesothelioma [12]. This study showed a benefit in median survival of 5 months and an increase in the response rate of 10% [11]. Ever since, other phase II studies were proposed but their benefit is still limited [13, 14]. Based on this data, for unresectable patients the OS is 55% at 12 months with a median survival of 13.1 months [11, 13].

Pleural mesothelioma which is more common and represents the model of choice for the treatment of peritoneal mesothelioma has also benefitted from phase III studies analyzing the addition of a targeted therapy (Bevacizumab) [15] and phase II trials proposing immunotherapy (Anetumumab) and other novel therapies [16].

By contrast, peritoneal mesothelioma has benefitted from very few systemic chemotherapy studies but was the setting of choice for testing intraperitoneal administration of chemotherapy either as early postoperative intraperitoneal chemotherapy [17] or as neoadjuvant intraperitoneal chemotherapy [18]. Both studies offered promising results suggesting a particular sensitivity of MPM to intraperitoneal administration.

Pressurized Intra-Peritoneal Aerosol Chemotherapy (PIPAC) has recently been developed and shows interesting results in the neoadjuvant context of several peritoneal carcinomatoses while producing little toxicity [19, 20]. PIPAC is a modality of repeated administration of intraperitoneal chemotherapy during laparoscopy using aerosols at the pressure of the capnoperitoneum (12 mmHg). Data from *ex-vivo, in-vivo* and human studies demonstrated a higher local drug bioavailability when compared to liquid IP chemotherapy [21]. PIPAC was tested in the setting of malignant mesothelioma showing encouraging results. The safety was evaluated on 29 patients out of whom 7 were assigned to alternating systemic and PIPAC regimens [22].

While neoadjuvant therapy for patients with unresectable MPM needs improvement [23], intraperitoneal drug delivery seems the option of choice for treatment intensification. The aim of this trial is to test an intensification of the neoadjuvant treatment in these patients using modern intraperitoneal drug delivery methods (PIPAC).

## Materials and methods

### Study setting

This phase II clinical trial is a national multicenter randomized study. It will be conducted in 10 sites, all specialized in the treatment of peritoneal carcinomatosis. The participating centers are part of the RENAPE (National Network for the management of the rare peritoneal tumors) network and they have extensive experience in the treatment of MPM. The recruitment period will last about 36 months, and each patient will be followed-up for 2 years after the end of treatment.

### Study design

The study is a phase II multicenter randomized trial evaluating the association of PIPAC and systemic chemotherapy vs. systemic chemotherapy alone as first-line treatment of MPM. Patients will be randomized with a 2:1 ratio using a minimization technique. Stratification will be performed according to histology (epithelioid vs. sarcomatoid and biphasic), presence of extraperitoneal disease and center.

### Objectives

The primary objective of the study will be to evaluate overall survival (OS). The secondary objectives will be to evaluate progression-free survival (PFS), safety, compliance, feasibility, conversion to resectability, histological response to treatment and quality of life.

Ancillary studies will assess the role of MSI status, EGFR and Ras mutations as predictive of OS and PFS, the role of mesothelin, calretinin and CA-125 as predictive factors of tumor response and will identify new imaging biomarkers of tumor response using radiomics.

### Inclusion and non-inclusion criteria

The study will include adult patients aged 18 to 75 years with histologically-confirmed diagnosis of peritoneal malignant mesothelioma, of performance status ≥2. Patients will not have received a previous treatment (medical or surgical). Their peritoneal carcinomatosis index (PCI) will be >27 or at least ≤4 on the small bowel with serosal involvement, i.e. contraindication for CRS because preserving a minimum length of 1.5 meters of uninvolved small bowel would not be possible. Patients presenting with any contraindication to chemotherapy, radiotherapy or to repeated laparoscopy will not be included in the study. Limited extra-peritoneal disease will be allowed (mediastinal and retroperitoneal lymph nodes, oligo metastatic lung disease). Patients with massive refractory ascites of more than 10 L per month will also be excluded. Exclusion criteria also include symptomatic cardiac or coronary insufficiency, severe renal insufficiency, progressive active infection or any other severe medical condition, intestinal occlusion non resolutive under medical treatment, previous cancer treated in the two preceding years except *in situ* cervical carcinoma or basocellular/spinocellular carcinoma, previous surgery for which laparoscopy was not feasible, and pregnancy or breast-feeding. Persons deprived of liberty or under guardianship, incapable of giving consent, or presenting with any psychological, familial, sociological or geographical condition which could interfere with compliance to the study protocol or follow-up will not be included in the study.

The sample size was determined based using a one-sample log-rank test (a two-sided 5% significance level and an 81% power). To detect a median OS time of 24 months in the experimental group with a median OS time of the historic control group of 14 months, accounting for 10% of patients lost-to follow-up, 66 patients are required, 22 in the control group and 44 in the experimental group. Subjects will be accrued for a period of 36 months and have a minimum follow-up period of 24 months after the last subject is added. It is assumed that the survival time distributions of both groups are approximated reasonably well by the Weibull distribution with a shape parameter of 1. The above gives an expected number of events of 28 in the experimental arm.

### Interventions

Only patients who signed the informed consent and completed all initial assessments exams to validate all inclusion and non-inclusion criteria will be included in the study. Patients will undergo a CT-scan, DW-MRI, PET-scan and laparoscopic initial staging before randomization. They will then be randomized (2:1) into two arms.

#### Randomization

Randomization will be centralized and performed using an e-CRF at the Biometrics Unit (CTD INCa) of the sponsor, the Montpellier Cancer Institute (ICM). The procedure of use of the e-CRF and inclusion will be given to all investigators at the opening of each investigating center. An identification number will be assigned to each patient, which will be retained for the whole trial duration.

#### Control arm

Patients in the control arm will receive standard systemic chemotherapy, Pemetrexed 500 mg/m^2^, IV 10 minutes, then cisplatin 75 mg/m^2^, IV 1 hour, 30 minutes after completion of the Pemetrexed. Patients will receive 6 cycles, every 3 weeks (day 1=day 22). Patients will receive adequate anti-emetic treatment and appropriate hydration prior and/or after receiving cisplatin.

#### Experimental arm

**Figure 1:**
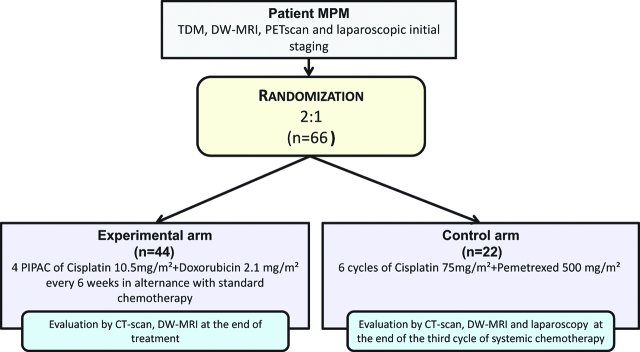
Consort flowchart of the study.

**Figure 2:**
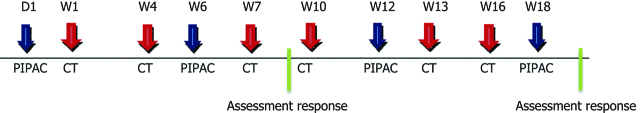
Therapeutic schedule of the experimental arm.

Patients in the experimental arm will receive a total of 4 PIPAC administrations (cisplatin 10.5 mg/m^2^ plus doxorubicin 2.1 mg/m^2^, 12 mmHg CO_2_) [24] every 6 weeks, and 6 cycles of standard chemotherapy (same chemotherapeutic combination than for patients of the control arm). The therapeutic schedule will be composed of 3 cycles of 1 PIPAC CD administration followed with 2 chemotherapy cycles. A final PIPAC CD will be administered after the 3 cycles (Figures 1 and 2).

**Figure 3:**
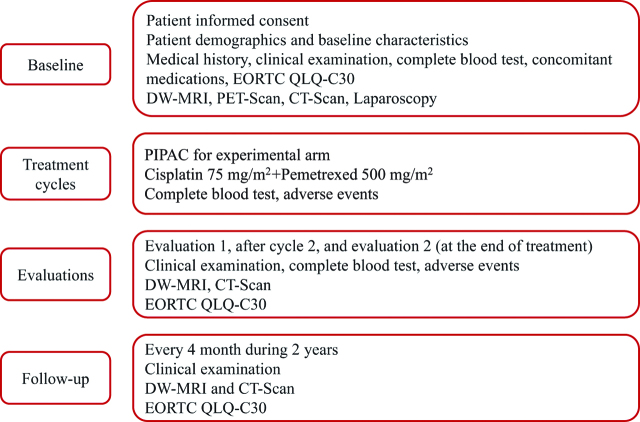
Data collection schedule.

The PIPAC procedure will be performed under general anesthesia. Antibiotic prophylaxis will also be administered to the patients of the experimental arm. The three steps of the experimental procedure will be as follows: (1) an open laparoscopy will be performed with inflation of the pneumoperitoneum, during laparoscopic exploration; (2) a PCI evaluation will systemically be performed and biopsies will be taken from each abdominal quadrant and analyzed for the evaluation of the peritoneal response score; (3) nebulization will be performed with a high pressure injector inserted in the peritoneal cavity, at 0.5 mL/sec at 20 bars to release chemotherapy as an aerosol in the abdomen. The closed system with zero flow will be maintained for 30 minutes at 37 °C. Toxic aerosols will be exhausted over a closed waste system.

After 3 cycles of systemic chemotherapy, patients of the two arms will be evaluated by a set of examinations including clinical examination, CT-scan, DW-MRI and quality of life. The final evaluation will take place at the end of treatment and will include the same examinations; a laparoscopic exploration will also be performed in the control arm to reevaluate resectability (Figure 3). Sample biopsies to estimate histological response will also be performed in those patients.

### Dose modifications

For the control arm, dose adjustments will be based on the nadir hematologic counts or maximum non-hematologic toxicity reported during the preceding chemotherapy cycle. Treatment may be delayed to allow sufficient recovery. PIPAC administration premature arrest in the experimental arm will be considered if grade III hematologic toxicities or grade III asthenia are reported after the first administration. PIPAC administration will also be stopped in case of surgical adverse event such as chemical peritonitis, important adhesions, bowel wound. Dose reduction of the systemic chemotherapy will be the same than for patients in the control arm.

Any dose modification or delay and its cause will be reported for patients in the control or experimental arms.

### Endpoints

The primary endpoint will be overall survival (OS) defined as the time from randomization to death from any cause. The secondary endpoints will include PFS defined as the time from randomization to any progression or death, described with its median and the 1 and 2-year PFS rates. Progression will be defined as any clinical or radiological change in the patient’s status: disease-related severe symptoms such as pre-occlusive or occlusive episodes requiring treatment, increased ascites requiring repeated evaluating punctions, new radiological targets or significant increase of existing lesions. Safety as well as complications related to PIPAC administration will be assessed according to the CTCAE (Common Terminology Criteria for Adverse Events) v.5.0. Surgical complications will also be graded according to the Clavien Dindo classification. Compliance to the study treatment will be assessed by the percentage of patients in the experimental arm who will have completed the whole treatment, i.e. the 4 PIPAC CD administrations and the 6 chemotherapy cycles. The conversion to resectability rate will be the percentage of patients eligible for CRS plus HIPEC in each arm, at the end of the treatment. Patients will be considered resectable if the preservation of at least 1.5 meters of small bowel and of at least 2 meters of lower gastrointestinal tube is possible in case of complete cytoreduction. The histological response to treatment will be assessed comparing the peritoneal regression grading score (PRGS) score [25, 26] of the samples at the last laparoscopic evaluation in the standard arm or the last PIPAC in the experimental arm compared to the initial histologic aspect. Last, quality of life will be assessed using the EORTC QLQ-C30 questionnaire.

### Statistical analyses

All analyses will be detailed in and performed according to a statistical analysis plan written before database lock. Populations will be defined as: intention-to-treat (ITT) patients, i.e. all randomized patients; per-protocol (PP) population, i.e. all eligible and evaluable patients (patients randomized and treated, with the two evaluations performed); safety population, i.e. all patients who received at least one dose of treatment. All statistical analyses will be performed on the ITT population, efficacy analyses on the PP population, and safety analyses on the safety population. Descriptive analyses will be performed using medians and ranges for continuous parameters, and frequencies and percentages for categorical variables. Baseline characteristics of randomized patients in each arm will be compared using the Kruskal-Wallis or Wilcoxon tests for continuous variables, or the [2 or Fisher exact tests for categorical variables. The median follow-up will be calculated using the reverse Kaplan-Meier method with its 95% confidence interval (95% CI]. All event-free survival (OS, PFS) will be estimated using the Kaplan-Meier method, and then described using medians and rates with their associated 95% CIs. Survival curves will be compared using Log-rank test. All toxicities will be described by arm. Analysis of the EORTC QLQ-C30 questionnaire will be performed in accordance with the EORTC guidelines. Exploratory analyses will be performed using the Time to definitive deterioration in quality of life, with the use of a 10-point minimal clinically important difference, analyzed with the use of the Kaplan–Meier method and the log-rank test. All statistical tests will be two-sided and a p-value <0.05 will be considered significant. The statistical analyses will be performed with the Stata v13.0 software.

### Ancillary studies

Biomarkers have been studied in the treatment of mesothelioma but not in the context of intensified first-line treatment. An update of the potential predictors of OS and response to treatment will be performed. The ancillary study will aim to detect the role of EGFR and Ras mutations in predicting OS and PFS, the role of mesothelin, calretinin and CA-125 in predicting response to treatment, and the definition of new imaging biomarkers of tumor response to treatment with the aid of radiomics.

### Safety

Every adverse event will be recorded on the corresponding page of the case report form (e-CRF). It will be documented, monitored and followed until the patient has recovered or until the safety follow-up visit is performed at 30 days after withdrawal of the investigational product. The severity or grade of all adverse events will be evaluated by the investigator following the NCI-CTCAE classification version 5.0.

For every serious adverse event (SAE), the investigator and the sponsor will evaluate separately the possible causal relationship to the investigational product. Every SAE, expected or unexpected, occurring during the study period, will be notified to the sponsor without any delay, using the “Serious Adverse Event Notification Form”.

### Data collection and management

Data collected will include the following: patient demographics and baseline characteristics, and medical preoperative history (age, weight, BMI, ASA, ECOG performance status cancer treatment, surgical history, comorbid conditions, indication for surgery, baseline quality of life), clinical examination (ECOG and vital signs, i.e. heart rate, blood pressure and weight), surgical data (accessibility, presence of adhesions, trocar placement, intraoperative incidents, operative time, presence and quantity of ascites at each laparoscopy, localization and number of biopsies), concomitant medications at baseline, postoperative assessments (toxicities, postoperative complications) and follow-up assessments (histological response, OS, PFS, recurrence and recurrence type, extra-peritoneal metastatic disease, feasibility of CRS, intraoperative and postoperative outcomes of CRS, quality of life).

Data will be collected directly by the identified and declared persons of each center and reported to the sponsor in an electronic Case Report Form (e-CRF) using the CSOnline software. All study documents and source documentation for the e-CRF will be kept by the investigators at the investigational sites according to the regulatory requirements.

### Study monitoring

Study monitoring will be conducted by a clinical research associate to verify the compliance with the study requirements and Good Clinical Practice (GCP) guidelines. Monitoring will be decided following a risk-based approach and will be performed in accordance with the GCP guidelines.

### Quality assurance

Data will be controlled and validated according to specific procedures. At the end of the study and once all the eCRF data are validated, the investigator will be logging to sign all the pages and validate the data entered for each patient. The sponsor will create and send an electronic copy (PDF file) to the investigator. This copy must be printed and signed by the investigator, to be archived at the investigator’s site.

### Regulatory and ethical compliance

All procedures and any consideration regarding the protection of human subjects will be carried out in accordance with the protocol, the GCPs, the ICH Guidelines, the ethical principles as detailed in the Declaration of Helsinki, and with all applicable regulatory requirements.

The protocol and informed consent form will be submitted for approval to the French National Security Agency of Medicines and Health Products (ANSM) and the ethical and Protection of Persons Committee (CPP) before study start.

Informed consent will be obtained for each patient prior to initiating any trial procedures, in accordance with the regulatory and ethical requirements. A copy of the signed informed consent will be given to each patient, and another will be retained in the investigator’s trial records.

All confidential information reported in the study documents will be held by the investigators in confidence. They will be entered into a database by the sponsor in accordance with the French law “Loi Informatique et Liberté” and with the European General Data Protection Regulation (GDPR 2016–679).

### Trial registration

The protocol has been registered on clinicaltrials.gov and assigned the following number: NCT03574493 EudraCT number 2019–001515-23 [27].

### Results dissemination

The results of the study will be presented in international surgical oncology meetings and will be published in peer-reviewed journals.

#### Roles and responsibilities

MESOTIP is a highly collaborative and integrated project with deliverable and assigned tasks and responsibilities depending on all study partners. The management of the project will be performed by the study sponsor and coordinator, the Montpellier Cancer Institute.

#### Study management committee

The study management committee will include individuals responsible for daily management of the study, i.e. the principal investigator, identified co-investigators or collaborators, and one representative of the clinical research department, one methodologist, the pharmacovigilance expert and a project manager and clinical research associate. The committee has approved the final version of the protocol before its submission to regulatory committees, will review the study progress and will be responsible for the protocol changes if required.

#### Investigators

The investigators will be responsible for the accuracy, completeness, legibility and timeliness of the data reported in all required reports. They will keep the trial documents as specified in the Essential Documents for the Conduct of a Clinical Trial, as required by the applicable regulatory requirements (International Council for Harmonization of Technical Requirements for Pharmaceuticals for Human Use, ICH). He will keep records, including the identity of all participating subjects, all original signed informed consent forms, SAE forms, source documents, and detailed records of treatment disposition.

#### Independent data and safety monitoring board

The independent data and safety monitoring board (DSMB) will have an advisory role and provide its opinion concerning all safety issues related to the study or study treatment. He will advise the sponsor to continue, modify or stop the study. The DSMB will be composed of at least 3 experts in clinical research and/or in the study indication (a methodologist, a medical oncologist and a surgical oncologist), will not be implicated in the study protocol, and are not allowed to have any financial interests in the study. They will sign a financial disclosure form and a confidentiality agreement form before attending the board. The members will meet at 6 months after inclusion of the 12th patient of the experimental arm; an annual meeting to evaluate safety and reliability of the trial is also scheduled.

### Network

All 10 participating centers are part of the RENAPE (Réseau National de Prise en Charge des Tumeurs Rares du Péritoine), the French national network for the management of rare peritoneal tumors, established in 2008 at the initiative of the French National Cancer Institute (INCa). RENAPE works in close collaboration with the AMARAPE, the Rare Peritoneal Tumor Patients.

## Discussion

The present protocol is the first trial to propose PIPAC combined to systemic chemotherapy as an intensification regiment in the first line treatment of unresectable peritoneal cancer patients. The patients included in the protocol will have a histologically proven diagnosis of MPM, a rare condition in which prospective trials are seldom initiated due to the difficulty of recruitment. Indeed, no other ongoing front line trial in MPM is currently listed on clinical-trials.gov [25]. The RENAPE network will facilitate the implementation of the trial, and thus patient recruitment, in France [26].

Our hypothesis is that intensification of the standard systemic treatment using PIPAC CD will lead to better results for the patients in the experimental arm. Overall survival is the primary endpoint of this phase II trial as response to treatment is difficult to evaluate in the setting of peritoneal metastases using the RECIST criteria. Previous trials in the field were more frequently performed in the pleural setting and surrogates of RECIST criteria were used such as pleural thickness and pleural effusion [13, 14, 15]. However this strategy is not standardized and it is less applicable to the peritoneal setting where the quantity of ascites is hard to determine and the presentation of the disease is heterogeneous. Nevertheless, overall survival may also be associated with confounding factors, usually related to the unequal availability of supportive care in different regions of the country. The participating centers provide trained teams of supportive care with homogenous practices. This aspect is important as survival for the standard arm is expected to be of 13 months [11, 13].

The dose used in this trial is the recommended dose after a phase I trial. Although the combination Cisplatin 10.5 mg/m² and Doxorubicin 2.1 mg/m² was shown to have limited toxicities [24], larger cohort data were not published yet.

The outcomes will be evaluated in a standardized manner. All toxicities as well as surgical complications will be defined according to the CTCAE v5.0. A recent publication has already shown that 90 days morbidity is better evaluated with CTCAE v5.0 than with the Clavien Dindo classification in patients who underwent CRS and hyperthermic intraperitoneal chemotherapy [29]. As PIPAC is a drug delivery method, it can be inferred that this classification is also superior to the Clavien Dindo classification [28]. However, the latter will also be present in the CRF for comparative purposes.

The results of this study will impact a population of patients affected by a rare disease and considered initially unresectable who are currently treated with a combination of drugs that has not seen any progress in the last fifteen years.
